# Importance of Maternal Nutrition in the First 1,000 Days of Life and Its Effects on Child Development: A Narrative Review

**DOI:** 10.7759/cureus.30083

**Published:** 2022-10-08

**Authors:** Akanksha Likhar, Manoj S Patil

**Affiliations:** 1 School of Epidemiology and Public Health, Jawaharlal Nehru Medical College, Datta Meghe Institute of Medical Sciences (DMIMS), Wardha, IND; 2 Research and Development, Jawaharlal Nehru Medical College, Datta Meghe Institute of Medical Sciences (DMIMS), Wardha, IND

**Keywords:** pregnancy, postpartum, maternal during conception, child changes over time, child nutrition, child growth and development, first 1000 days of life

## Abstract

Maternal nutrition needs to be addressed during pregnancy for the child’s first 1,000 days of life, or roughly between conception and a child’s second birthday. The infant requires just breast milk for the first six months of life. The production of breastmilk and its nutritional value is essentially unaffected by maternal privation. The child’s health suffers when the mother’s diet and health are impaired. This review aims to discuss the importance of pregnant women’s nutrition and how it impacts the development and expansion of a child during this critical period of development, which is supported by the most recent literature. Throughout the child’s growth in the mother’s womb and outside, four distinct stages have been identified: (1) nine months to zero months: pregnancy; (2) zero to six months: breastfeeding; (3) six to 12 months: introduction of solid food; and (4) >12 months: transition to family diet, appreciation of nutritious food offered within each period for the child’s development. Moreover, there is a strong link between nutrition, well-being, and learning. The nutritional intake of infants, children, and adolescents maintains the body weight and is sufficient to sustain their normal growth and development. One of the crucial factors influencing a child’s development is nutrition. Rapid growth occurs during infancy. Compared to other growth phases, this phase has the largest relative energy and food needs for body size.

## Introduction and background

The 1,000 days hold both great possibility and great fragility in a child’s life. The ability of a child to develop, learn, and thrive is significantly impacted by how well or how poorly mothers and children are fed and cared for throughout this period. This is because the developing brain of a child grows and develops throughout the first 1,000 days. This is also the period when the foundations for their long-term health are set [1]. Children’s brains can create 1,000 new neural connections every second throughout this time. The connections that a three-year-old brain forms provide the foundation for their future because it is twice as busy as an adult’s brain [2]. Achieving both physical and mental wellness is a lifetime goal. A person’s first 1,000 days, or the period from conception to age two, are the most crucial for the development of their body, brain, metabolism, and immune system. Malnutrition is associated with numerous health problems, such as obesity and impaired growth [3]. Prenatally, the seeds of a baby’s health are sown, and they develop throughout early development and the effects of the first 1,000 days on psychological and physical health [3]. Research indicates that a pregnant woman’s well-being, diet, and stress levels likely affect the development of her unborn child. After birth, the child’s physical environment, nutrition, and relationships may have a long-term effect on their well-being and health [4]. During this stage, a child’s ability to grow can be affected by maternal and child nutrition and health. According to specialists, a child’s developing brain and body suffer irreparable damage from insufficient nutrition in the first 1,000 days of life [5]. The feeding and care needed by the expectant mother throughout the first trimester of her pregnancy impact the first 1,000 days of the pregnancy itself. Early pregnancy discovery is crucial to ensure the window of opportunity presented as of the first 1,000 days is used to give the child and mother a good future. The window of opportunity to remedy some deficiencies shortens as it ages, becoming a challenge for a grown-up or older child to handle the health difficulties brought on by a deficit. However, some deficiencies can be partially remedied later in life [5].

## Review

Methodology

We conducted a search of electronic databases with a specific goal in mind to find literature, a quest for reliable summaries, and various sources, including books, that have detailed conceptual and theoretical studies, handbooks, and gray literature. We examined what medical and social experts have been working on to discover the parameters that allow children to achieve optimal health and development. A literature search was done systematically on PubMed, EMBASE, Google Scholar, and others. Major search terms were the first 1,000 days of life, maternal nutrition, child growth and development, solid feeding, breastfeeding, and diets of the child. Inclusion criteria for the study included mothers in the stages of preconception, during the pregnancy and postpartum period, and six months, nine months, and 12 months of children. Exclusion criteria were studies involving unmarried women, teenagers, and adolescents.

Discussion

Why Nutrition Matters?

A child’s first 1,000 days of life are the most delicate and important for laying the foundation for their healthy growth and development. Changes in the results of birth and growth and newborn stunting have been demonstrated by dietary support for the first 1,000 of life. Poor maternal and child health outcomes, particularly poor birth outcomes, are linked to inadequate maternal nutrition during conception, during pregnancy, and after delivery [6]. The development of the individual as well as the nation depends on nutrition. The evidence presented here adds to the body of research showing that various developmental goals are fundamentally influenced by good nutrition. Addressing malnutrition must be the top priority of the post-2015 sustainable development agenda [7]. A child’s ability to develop, learn, and thrive fundamentally depends upon good nutrition during pregnancy and the early years of life [8]. According to research on how children develop, healthy eating; consistent, responsive interactions with caregivers; and caring settings are the three fundamental supports that children need to succeed as people. The physical, social, emotional, and cognitive development can suffer when one or more of these supports are lacking, which can lead to the loss of possibilities that are every child’s birthright [9]. Several nutrients are crucial for the pregnancy-induced growth of the brain. These consist of specific lipids, protein, copper, folate, zinc, iodine, iron, and copper [9]. Children who receive proper nutrition in the first 1,000 days have a higher likelihood of birthing at a healthy weight; have a reduced risk of developing a number of illnesses and disorders, such as obesity and type 2 diabetes; become better learners with fewer behavioral issues in kindergarten; and benefit from better health and financial stability [10].

Effects of Nutrition on Child Development

Complex processes such as growth and development call for a proper ratio of nutrients [11]. The health of a mother and her child are intertwined during the first 1,000 days. Women must receive the nutrition, attention, and assistance they need to ensure a healthy future for themselves and their children [12]. Prior to conception, during pregnancy, and during nursing, a well-planned mother’s diet is crucial [13]. A reversal can be exceedingly challenging beyond age two, thus optimal diet and correcting nutritional deficits during the early years are crucial [14]. To enhance children’s growth, health, and survival, however, the World Health Organization (WHO) and United Nations International Children's Emergency Fund (UNICEF) created the Global Strategy for Infant and Young Child Feeding [14]. The physical and mental development of a child may be hindered if a woman is undernourished when she is pregnant or during the first two years of the child’s life. The child will be affected by this for the rest of his or her life and it cannot be undone when the youngster is older. Children have the right to a loving, safe environment, wholesome food, and essential medical treatment to keep them healthy, foster growth, and promote development [15]. During this complex stage of life, there are numerous factors that contribute to growth, including diet, genetic and epigenetic factors, and hormone regulation [16]. All necessary nutrients should be obtained to encourage a wholesome pregnancy and promote optimal development. Eight vital nutrients play a specific role throughout the first 1,000 days of life, namely, carotenoids (lutein + zeaxanthin), choline, folate, iodine, iron, omega-3 fatty acids, and vitamin D. Other nutrients for maternal, newborn, and toddler health include magnesium, vitamin A, vitamins B, and other trace minerals [17]. While essential for fetal neurodevelopment, folic acid and iodine are frequently deficient in pregnant women’s diets [18]. Stunting or poor fetal growth during the first two years of life causes permanent harm, including reduced offspring birthweight, shorter adult height, lower adult income, and lower adult education [19]. Table 1 lists the four stages when development occurs in the life of a child.

**Table 1:** Child development stages.

Serial number	Stages
1	Nine months to zero months: pregnancy
2	Zero to six months: breastfeeding
3	Six to 12 months: introduction of solid food
4	>12 months: transition to the family diet

Nine Months to Zero Months: Pregnancy

A healthy, balanced diet before conception and during pregnancy assists the growing fetus in being nourished and helps the mother create the best bodily reserves in anticipation of breastfeeding [20]. According to the general population’s dietary recommendations, pregnant women should eat a balanced diet. Late in pregnancy, they should only slightly increase their calorie intake from what is advised for women who are not pregnant, no more than 10% [21]. For the health of the fetus, crucial considerations include the mother’s weight, eating habits, and nutritional condition before and throughout pregnancy. Pregnancy complications relating to conception, placenta, embryo, fetal development, fetal growth, and perinatal problems, as well as maternal and paternal fertility, are before and during pregnancy, caused by both inadequate and excessive nutrition and weight, with suboptimal pregnancy outcomes for the mother and child [22]. Eating a variety of foods in the recommended quantities is a smart step toward maintaining good health [23]. During pregnancy, protein forms and repairs blood and uterine muscle tissue as well as the baby’s tissue. Iron acts as the core element for the protein hemoglobin, which transports oxygen from red blood cells to tissues. Iron is necessary to the baby’s blood supply during pregnancy as the amount of blood in the body rises. The development of the teeth and bones of the infant, as well as wound healing, depends on vitamin C. The baby’s heart, muscles, bones, teeth, and nerve function develop due to calcium, which is also important for fluid control. The brain and spine of an infant require folic acid. During pregnancy, folic acid also aids in blood production. A severe birth problem of a baby’s brain or spine, neural tube defects (NTD), can be avoided with enough folic acid in the diet. With adequate folic acid, NTDs can be prevented in 70% of cases [24].

Zero to Six Months: Breastfeeding

Breast milk provides a newborn with the ideal dietary foundation for the first six months of life because it is specially formulated to meet the needs. All the nutrients a newborn needs are in the proper amounts in breast milk. Additionally, colostrum aids in the early days of immunity building and infection prevention. Breast milk keeps changing as they develop to meet evolving nutritional needs [25]. Breastfeeding helps balance nutrition throughout the first six months, which is a window of opportunity unmatched by any other first food. It guarantees that the child receives the finest start in life. All newborns should breastfeed exclusively until they are six months old, according to pediatricians. Mothers and relatives are not required to give the infant glucose, jaggery, sugar, plain water, or honey during this stage. Breastmilk, which contains all the nutrients a baby needs to grow and develop, is a healthy and complete diet for any infant under the age of six months [26]. Human milk can meet all nutritional requirements in the first six months of life, with the exception of vitamin D, according to pediatricians. Infants who are breastfed or get less than 27 ounces of formula per day should be given a vitamin D supplement that contains at least 400 IU of vitamin D as soon as possible after delivery. Most newborns have enough iron reserves to last for the first six months of life. Omega 3 DHA is present in human milk and the majority of commercial baby formulae [27].

Six to 12 Months: Introduction of Solid Food

Offering nutrient-rich meals helps the baby develop wholesome eating habits for the future, even if breastmilk and formula provide the majority of the nourishment until the child is a year old. Additionally, there are a few nutrient shortages that may need to be supplied beginning at roughly six months [28]. When it comes time to introduce solids, the majority of the child’s calories and nutrients come from breast milk or formula. Instead, solid foods should be viewed as bonus food [29]. Many parents find it difficult and perplexing to introduce solid and semi-solid foods to their infant’s diet. The age of the child, their hunger, and their rate of growth are all aspects to consider when introducing solid foods. The American Academy of Paediatrics (AAP) advises that semi-solid foods start when an infant is six months old because they represent a big transition. This age often corresponds with the neuromuscular maturation required for eating solid foods. When to introduce solid foods to a toddler depends on a variety of factors, including age, hunger, and growth rate.

Grain products: When first giving solid foods to a child, simple grains like rice cereal should be considered. Grains provide extra iron that is necessary for healthy growth and development. Wheat products should be introduced last because they are more allergic.

Fruit: Fruits that are pureed, unadorned, or ripe, such as mashed bananas, peaches, or applesauce, can be considered. Puree the fruit after adding breast milk or baby formula. Citrus fruits should be avoided due to their high acidity during the first year of life, and desserts made with fruit should not contain extra sugar. Desserts contain extra calories that are not required and could increase weight and obesity. Moreover, 100% fruit juices can be introduced to the baby when they are seven months old and can sip from a cup. Before providing 100% fruit juice to a baby, it is vital to filter the pulp or dilute it with water. Sugary beverages like sports drinks, soda, and tea should be avoided as these might lead to tooth damage and add unneeded calories to the diet.

Vegetables: They can be pureed similarly to fruits by adding breast milk or newborn formula. Vegetables should not be salted because doing so could put a burden on a baby’s kidneys.

Protein: Using breast milk or infant formula, puree proteins like chicken, beef, pig, tofu, or beans in a way similar to how fruits and vegetables are prepared [30].

>12 Months: Transition to Family Diet

**Figure 1:**
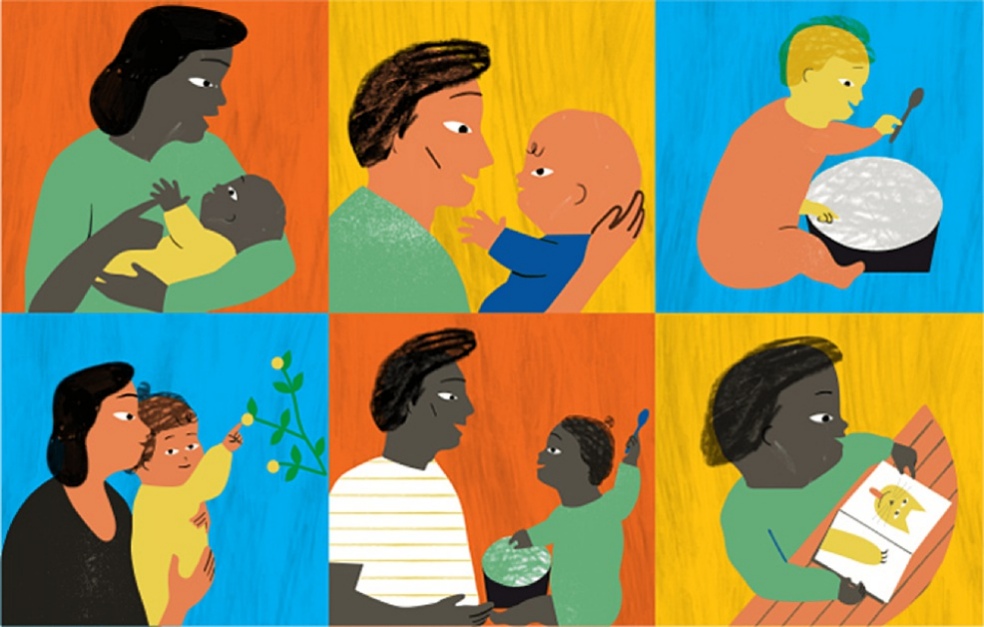
Six stages from newborn to two years: the first 1,000 days of life. Available at https://blogs.unicef.org/jamaica/the-first-1000-days/ [34].

During toddler years, growth slows down slightly, but eating remains a major necessity. Additionally, now is the time for parents to change course, do away with bottles, and usher in a new era wherein children eat and drink on their own [31]. To provide adequate nourishment once a youngster begins to eat a variety of meals when starting solids. Despite their tendency to be fussy eaters, we should encourage young children to eat a range of meals. It can be essential to try a food type again with different meals before a youngster will accept it, often eight to 15 times. As they grow, children normally adapt by changing their food consumption on their own. A child’s need for protein, vitamins, and minerals increases as they become older, just like their demands for energy [32]. Approximately by the age of 12 months, healthy growth should be encouraged, the AAP recommends consuming 1,000 calories, 700 mg of calcium, 600 IU of vitamin D, and 7 mg of iron daily [33] (Figure 1).

## Conclusions

A child’s future health depends on proper nutrition during the first 1,000 days of life, as well as during pregnancy and the early years of life. According to research, the diet of the mother, weight, and lifestyle choices might affect the infant’s immune system, organ development, and metabolism. Building a relationship with parents or other caregivers in a secure and loving setting, eating the right foods (and providing a nurturing environment for both mother and baby), and engaging in regular, stimulating play are essential components of healthy child development. Children who experience the positive effects of the first 1,000 days can earn up to 20% more as adults than their peers and are more likely to start their own families.
